# The Characteristics of the Metabolomic Profile in Patients with Parkinson’s Disease and Vascular Parkinsonism

**DOI:** 10.32607/actanaturae.27511

**Published:** 2024

**Authors:** E. V. Predtechenskaya, A. D. Rogachev, P. M. Melnikova

**Affiliations:** Novosibirsk State University, Novosibirsk, 630090 Russian Federation

**Keywords:** metabolomics, mass spectrometry, biomarker, Parkinson’s disease, vascular parkinsonism

## Abstract

The gradually increasing age of the world population implies that the prevalence of neurodegenerative diseases also continues to rise. These diseases are characterized by a progressive loss of cognitive and motor functions. Parkinson’s disease, which involves the gradual death of specialized neural tissue, is a striking example of a neurodegenerative process. The pathomorphological analysis shows that chronic cerebral ischemia is accompanied by extensive complex neurodegeneration; parkinsonism is its clinical manifestation in 20–30% of cases. Although Parkinson’s disease and vascular parkinsonism are similar, these two pathologies have fundamentally different etiopathogeneses. But their set of differential diagnosis traits is confined to some features of the neurological status. There currently exist no diagnostic markers for individual neurodegenerative pathologies or the neurodegeneration phenomenon in general. Metabolomic profiling can be a promising means for finding a unique “fingerprint” of the disease. Identifying the biomarkers of various neurodegenerative diseases will help shorten the time to the diagnosis, forecast the course of the disease, and personalize the therapeutic approach. This review summarizes and compares the current concepts of metabolomics research into Parkinson’s disease and vascular parkinsonism, as well as the respective animal models.

## INTRODUCTION

Neurodegenerative diseases are among the most common causes of disability in developed countries. The growing wellbeing of the planet’s population has come with increasing human life expectancy and higher demands on the quality of life. Neurodegeneration is also an integral part of aging, being as it is responsible for the erosion in human functional ability and loss of cognitive capacity. Parkinson’s disease (PD) is a multisystem neurodegenerative disease with motor (hypokinesia, tremor, and rigidity) and non-motor symptoms (cognitive impairment). The pathogenesis profile of PD is mostly characterized by the destruction of dopaminergic neurons in the substantia nigra. But many different structures of the central nervous system are also caught up in it. Such widespread neurodegeneration leads to pronounced neurological deficit, which manifests itself as significant social and household disorientation of patients [1, 2, 3].

Vascular parkinsonism (VP) is clinically described as symmetric lower-body parkinsonism, which is characterized by postural instability, freezing of gait, and frequent falls [4]. However, there exists no specific clinical symptom that would be pathognomonic of VP. Various chronic cerebrovascular diseases (CVDs) are the pathophysiological foundation of VP, microangiopathy induced by chronic hypertension being the most common one [5]. During brain imaging, VP is shown to be associated with white matter hyperintensities or multiple infarcts in the basal ganglia and subcortical regions [6, 7]. CVDs are characterized by the involvement of the whole brain tissue (neuronal and non-neuronal) into degeneration, which implies that complex post-ischemic neurodegeneration follows [8].

Hence, although the impact of neurodegenerative processes on the life of our contemporaries is quite relevant, such diseases often remain difficult to diagnose. Currently, there are specific diagnostic laboratory markers for neither neurodegeneration in general nor neurodegenerative diseases in particular. Thus, PD and VP are differentially diagnosed in practice only according to the clinical findings, which is often insufficient.

This review focuses on the features of the changes in the metabolomic profile of patients with PD and VP. Metabolomic analysis is a promising research method that allows one to mine data on the biochemical changes taking place during a pathological process and identify potential biomarkers of the disease. Such data may both deepen the fundamental knowledge of the pathogenesis of parkinsonian disorders and propose potential diagnostic tools for practical application.

## METABOLOMIC STUDIES IN BIOMARKER SEARCH

Metabolomics is the systematic identification and quantitation of all the metabolites present in a biological system, which consists of numerous molecules that exhibit different physical and chemical properties and exist over an extensive dynamic range. The overall analysis of metabolites can improve our knowledge about the physiological, pathological and biochemical statuses of a being, information which can be further combined with chemical and informatics methods [9].

Metabolites are not only endogenous substances present in the body; they can also include products of the metabolism of pharmaceuticals, environmental chemicals, and such substances as the products of the interaction between the host organism and its gut microbiota. Even minor changes in endogenous and exogenous factors can affect metabolite levels. Hence, metabolomics has a great potential to help us identify the relationship existing between genetic, environmental, and physiological elements and certain pathological conditions [10].

Metabolomic studies can improve our understanding of the mechanisms of diseases and therapeutic effects; they can also enable us to predict individual disease progression.

## PATHOGENETIC PATHWAYS OF PARKINSON’S DISEASE

PD is based on progressive degeneration of the nigrostriatal dopaminergic pathway, with significant neuronal loss in the substantia nigra pars compacta and dopamine depletion. Along with disruption of the nigrostriatal dopaminergic system, in patients with PD neurodegeneration affects many groups of neurons residing in certain parts of the cerebral cortex, thalamus, brainstem, spinal cord, as well as in sympathetic and parasympathetic ganglia. This leads to the degeneration and death of both nigral neurons and the neurons located in extranigral areas [11]. According to Braak et al., neurodegeneration progression involves certain morphological stages: from the primary lesion of nuclei of the vagus nerve and the olfactory bulb to the gradual death of neurons in the substantia nigra pars compacta. This sequence is consistent with the development of clinical symptoms of PD ranging from autonomic disorders to motor and cognitive impairment [12]. It is noteworthy that early clinical signs of the disease occur only after 60–80% of substantia nigra neurons have been lost [13], which becomes responsible for the severity of the pathological process down the road.

The etiology and pathogenesis of PD still need thorough study; however, they are known to involve many predisposing factors (primarily genetic ones) and pathogenic pathways. The latter include:

(1) the formation of pathological specific α-synuclein in the form of Lewy bodies or Lewy neurites;

(2) oxidative stress associated with mitochondrial dysfunction;

(3) proteolytic stress caused by dysfunction of the ubiquitin–proteasome system; and

(4) local inflammation [11, 14].

Probably, none of the aforelisted mechanisms acts on its own; on the contrary, they mutually potentiate each other’s effect. Moreover, each of the aforementioned pathways can induce intracellular apoptosis, which is the final common mechanism of neuronal loss in PD.

The native α-synuclein molecule in the brain is mostly unfolded and has no well-defined tertiary structure. When interacting with negatively charged lipids such as phospholipids (components of cell membranes), α-synuclein (α-Syn) acquires a β-sheet-rich amyloid-like structure that is prone to aggregation [15]. In turn, formation of α-Syn inhibits potentiation in mitochondria, thus causing the dysfunction associated with complex I, a component of the electron transport chain [16]. For this and probably other reasons, patients with PD show multiple signs of oxidative stress in the substantia nigra. In particular, this is manifested as reduced levels of endogenous antioxidants (e.g., glutathione), while the levels of oxidation products of proteins, lipids, and DNA are significantly elevated. Hence, there appears to be a relationship between the theories of α-synuclein accumulation and mitochondrial stress [17].

Another important clue to the importance of mitochondria in disease pathogenesis is that many of the known genes causing familial PD are involved in mitochondrial homeostasis. These genes include the known *PINK1/Parkin *genes, which participate in the pathway regulating dysfunctional mitochondria: the process known as mitophagy [18].

Failures in protein clearance are also observed in patients with PD, along with mitochondrial dysfunction. Within cells, there are two central systems responsible for the removal of malfunctioning proteins: the ubiquitin–proteasome system and the autophagy lysosomal pathway. Monomeric α-Syn is normally cleared by both systems, and disruption of either of these mechanisms is implicated in the pathogenesis of PD, as it promotes the accumulation of defective proteins, including misfolded α-Syn [19].

## METABOLOMIC STUDIES OF PARKINSON’S DISEASE

The metabolome of patients with PD is studied using both non-targeted and targeted analytical approaches. The former involve large-scale metabolite screening followed by biomarker search among unknown metabolites, while the latter are based on the analysis and evaluation of a range of metabolites of interest, such as catecholamines, amino acids, purines, and urates. Most metabolomic studies rely on the analysis of cerebrospinal fluid (CSF) and blood, although other biological specimens such as patient urine, feces, or brain tissue were examined in some studies.

**Metabolomic studies of cerebrospinal fluid in Parkinson’s disease**

Irregularities in the CSF composition is directly related to pathological changes in the brain, making CSF one of the preferred specimens for neuropathologic research. Taking into account the marked depletion of nigrostriatal dopaminergic neurotransmission in patients with PD, by measuring the levels of dopamine and its metabolites, one could identify potential markers of the pathophysiological stage of the disease. However, such compounds can be reliably detected only in patients who are not on levodopa. Thus, a reduced level of dihydroxyphenylacetic acid (DOPAC) is one of the markers of changes in dopamine metabolism in PD [20]. Furthermore, it was demonstrated that DOPAC can be used as a marker of early stages of the disease [21]. Not only DOPAC, but also homovanillic acid (HVA, the major catabolite of dopamine) is regarded as a biomarker of PD; however, it currently is considered a less reliable marker of central dopamine metabolism compared to DOPAC [22]. Trupp et al. reported that the CSF level of 3-hydroxyisovaleric acid is reduced in patients with PD [23]. Interestingly, 3-hydroxyisovaleric acid is degraded by the same enzyme as tyrosine (levodopa precursor). The same study additionally reported that tryptophan and creatinine levels are decreased in PD patients.

Purines circulating in the CSF of patients with PD are also of interest. LeWitt et al. studied changes in the level of xanthine (a precursor of urates) in PD and demonstrated that the ratio of xanthine to homovanillic acid concentrations in CSF can be both a sign of the disease *per se *and a biomarker of the gravity of the patient’s condition [24].

An analysis of the CSF metabolome in patients with PD showed changes in the metabolism of glycine, serine, and threonine amino acids [25]. Differences in the contents of metabolites such as sarcosine and alpha-N-phenylacetyl-*L*-glutamine were found in the blood plasma and CSF of patients with PD compared to healthy donors. These compounds are involved in oxidative stress response in the metabolic pathways of sphingolipids, glycerophospholipids, and amino acids and can help in the early diagnosis of PD. The association of the oxidative stress metabolic pathways with PD is also supported by changes in the tricarboxylic acid profile, which is indicative of the development of mitochondrial dysfunction in this disease [26]. The same study revealed changes in the lipid profile in PD patients: an elevated level of medium- and long-chain fatty acids, as well as changes in diacylglycerol, phosphatidylcholine, and phosphatidylethanolamine metabolism.

**Metabolomic studies of blood plasma in Parkinson’s disease**

Metabolomic studies of blood plasma are becoming increasingly the preferred route thanks to the minimal invasiveness of sampling and the relative availability of blood specimens. Various amino acids, fatty acids, acylcarnitines, lipids, purines, organic acids, which are the components of the energy metabolic pathway, oxidative stress responses, and metabolic pathways specific only to PD, are considered as potential blood plasma biomarkers in PD (*Table 1*).

**Table 1:** Potential metabolomic markers of Parkinson’s disease

Biological matrix	Clinical stage of the disease	Patients receiving specialized therapy	Found biomarkers	Reference
CSF	n/d	No	DOPAC, HVA	[20]
CSF	n/d	No	DOPAC	[21]
CSF	n/d	n/d	3-Hydroxyisovaleric acid, tryptophan	[23]
CSF, blood plasma	Early stages (1–2 according to the Hoehn and Yahr scale)	No	Xanthine/HVA ratio (CSF); caffeine metabolites and inosine (blood plasma)	[24]
CSF	Different stages (1–4 according to the Hoehn and Yahr scale)	Yes	Glycine, serine, threonine	[25]
CSF	n/d	Yes	Profile of tricarboxylic acids, mediumand long-chain fatty acids, diacylglycerol, phosphatidylcholine, phosphatidylethanolamine	[26]
Blood plasma	Different stages (1–4 according to the Hoehn and Yahr scale)	Yes	Kynurenic acid, kynurenic acid/kynurenine ratio, quinolinic acid (late stages)	[27]
Blood plasma	n/d	Yes	3-Hydroxykynurenine/kynurenic acid ratio, 5-hydroxytryptophan	[28]
Blood plasma	n/d	Yes	Uric acid (PD, LRKK), hypoxanthine (PD)	[30]

Note: n/d – no data in the original article.

Chang et al. [27] described changes in the contents of kynurenine metabolites in PD: they considered these compounds a potential pool of disease biomarkers and discovered a novel therapeutic strategy, where kynurenic acid was additionally used or the quinolinic acid level was reduced using kynurenine- 3-monooxygenase inhibitors [27]. Moreover, Havelund et al. showed that kynurenine metabolism is also associated with the development of levodopa-induced dyskinesia, and that the elevated blood plasma ratio of 3-hydroxykynurenine to kynurenic acid can predict the potential progression of dyskinesia [28].

There are studies where urates are regarded as a promising biomarker of risk, diagnosis, and prognosis of PD. It was reported that the CSF and blood levels of urates are significantly reduced in patients with PD compared to the controls; high urate levels can be indicative of lower risk and slower disease progression. Elevated levels of these metabolites, which are important biogenic antioxidants, may help combat oxidative stress in the pathogenesis of PD. Various mechanisms have been proposed to explain the paradoxical effects of uric acid, but its significance as a causative, compensatory, or arbitrary risk factor remains unclear. High uric acid levels were shown to play an important role in preventing the involvement of dopaminergic cells in the pathophysiology of PD through its function as an endogenous antioxidant [29]. LeWitt et al. revealed changes in the profile of caffeine metabolites during the progression of PD, as well as a decline in the plasma level of inosine in patients with PD [24]. Differences in uric acid levels and purine profiles were also found in PD patients carrying a mutation in the *LRRK2 *gene and in healthy donors [30].

Changes in the bile acid profile can be a potential metabolomic marker of PD. For example, Shao et al. found increased levels of a number of bile acids (including microbiota-associated ones) in patients with PD [31]. Changes in the bile acid profile were shown both for patients carrying a mutation in the *LRRK2* gene and in patients with idiopathic PD along with changes in the purine base profile [32].

As mentioned previously, PD is a multifactorial disease; compelling epidemiologic data suggest a possible association between traumatic brain injury (TBI) and the onset of parkinsonian syndrome. Changes in the plasma levels of glutamate were observed by HPLC/MS in patients with TBI and those with PD, thus an indication of a possible “excitotoxic” role of glutamate in their pathogenesis [33].

The metabolomic approach can also identify biomolecular and metabolic changes affecting the onset and progression of the disease. Thus, changes in the spermidine metabolism and the N1,N8-acetylspermidine level can be a prognostic marker of PD progression, which may lead to a new strategy for delaying or slowing down the progression [34]. A strong correlation between the levels of alanine, methionine, serine, purine, a number of fatty acids, polyamines, and tryptophan metabolites and progression of PD was demonstrated [23, 35, 36].

Acylcarnitines can be potential markers of oxidative stress and mitochondrial dysfunction in patients with PD. Thus, changes in the acylcarnitine profile may be indicative of early stages of PD [37]. In addition, differences in the acylcarnitine profile were found in patients with PD and those with essential tremor [38].

**Metabolomic studies of Parkinson’s disease in experimental models**

Different types of animal models have been developed to study PD, but only a number of them have been used in metabolomic studies. For example, such models include α-Syn knockout, transgenic α-Syn, α-Syn overexpression, Park2 knockout, and toxicological models. The metabolic profile of the experimentally induced disease has been studied mainly in animal brain tissue, which better reflects pathophysiological changes but has obvious limitations in extrapolating similar processes to humans with PD.

Farmer et al. reported significant changes in the levels of lipids (belonging to the phosphatidylcholine and lysophosphatidylcholine classes) in brain tissue in the toxin (6-hydroxydopamine)-induced model of PD. These findings can be attributed to increased oxidative damage to lipids and also indicate that these molecules play an important structural and neurofunctional role [39].

Another study focusing on the metabolome of brain tissue, conducted in a model of PD induced by unilateral injection of preformed fibrils of α-synuclein into the olfactory bulb, showed dysregulation of taurine and the hypotaurine metabolism, bile acid synthesis, metabolism of glycine, serine, and threonine, as well as the tricarboxylic acid cycle, which correlated with the emergence and progression of pathologic α-Syn [40]. Theoretically, the emergence of these α-Syn aggregates is accompanied by the suppression of the metabolic pathways of glycine, serine, and threonine (in normal nervous tissue, these substances can be converted to creatine, which is a donor of phosphate groups for ATP).

Kim et al. showed a reduction in the levels of* L*-3,4-dihydroxyphenylalanine (levodopa) and dihydroxyphenylacetic acid in mice at the preclinical prodromal stage of PD induced by 1-methyl-4-phenyl- 1,2,3,6-tetrahydropyridine (MPTP) [41]. Theoretically, these changes can be regarded as biomarkers of the “presymptomatic” stage of PD. Other potential markers of PD can include 5’-methylthioadenosine, tetradecanoylcarnitine, phytosphingosine-1-P, ceramide d18:0/18:0, lysophosphatidylcholine 20:4(5Z,8Z,11Z,14Z),* L*-palmitoylcarnitine, tetracosanoylglycine, morphiceptin, and stearoylcarnitine; their levels were found to have changed when studying the midbrain in the MPTP-induced mouse model of PD [42].

Lu et al. identified the metabolites involved in oxidative stress, energy deficiency, and neuronal damage in goldfish with MPTP-induced PD using NMR [43]. The model was characterized by elevated levels of leucine, isoleucine, valine, and alanine amino acids, as well as alanylalanine, creatinine, myo-inositol, 18:2 fatty acid and total fatty acids, as well as simultaneous reduction in the levels of N-acetylaspartate, phosphocreatine, phosphocholine, betaine, glutamine, 3-hexenedioate, acetamide, malonate, isocitrate, scyllo- inositol, phosphatidylcholines, cholesterols, omega-3 fatty acids, and polyunsaturated fatty acids in the brain of goldfish. It was demonstrated by NMR that activity of the glutamate–glutamine cycle in the striatum of MPTP-treated mice was excessively high [44]. In the same mouse model, Pedro Amorim Neto et al. showed changes in the metabolic profile both in brain tissues and in peripheral structures such as the intestine using NMR [45]. Metabolite expression in blood, brain, colon, and feces specimens was deemed mostly indicative of inflammatory aspects, cytotoxicity, and mitochondrial dysfunction (oxidative stress and energy metabolism). A study conducted in mice showing symptoms of MPTP-induced PD and gut dysbiosis showed that biomarkers characteristic of such damage include 67 molecules associated with lipid and amino acid metabolism [46].

It is worth mentioning that the pharmacological effect of different anti-PD drugs in animal models can be controlled using metabolomics methods. Thus, the ability of various therapeutic agents to regulate the metabolism of amino acids, unsaturated fatty acids [47], purines, glycerophospholipids [48], as well as the neuroprotective effect of drugs through modulation of the gut microbiota–metabolite axis, has been demonstrated in MPTP-induced models of PD [49, 50, 51, 52].

## PATHOGENETIC PATHWAYS OF VASCULAR PARKINSONISM

Pathogenetic disorders leading to VP are primarily associated with cardiocerebrovascular risk factors, which include hypertension, hypercholesterolemia, cardiovascular diseases, type 2 diabetes mellitus, and advanced age [7]. These factors cause cerebrovascular disorders and affect the functioning of cerebral vessels (small vessel disease). Thus, arterial occlusion caused by the aforementioned factors would lead to various lesions in the basal ganglia and the pons, lacunar infarcts in the white matter, cerebral microangiopathy, disruption of endothelial tight junctions, and destruction of the BBB. Such disorders will be crucial in the pathogenesis of vascular ischemia [53]. In addition, small vessel changes such as gliosis, perivascular pallor, hyaline arteriolosclerosis, and especially enlarged perivascular spaces were observed in autopsy specimens from patients with VP [54].

There is mounting evidence that vascular risk factors contribute to the development of neurodegeneration. They affect the structure and function of cerebral vessels and associated cells; the so-called neurovascular unit. The neurovascular unit involves neurons, glia, as well as perivascular and vascular cells that work in close cooperation to maintain the homeostasis of the brain microenvironment. This structure regulates blood flow, controls exchange through the BBB, facilitates immune surveillance in the brain, and provides trophic support. Hemodynamic changes affect the structure of the neurovascular apparatus, leading to its dysfunction [53]. Pathomorphologically, this phenomenon will be characterized by a thickening of the vascular wall matrix, undesirable collagen accumulation, smooth muscle collapse, and vascular stenosis [55]. Damage to the neurovascular system alters the regulation of the cerebral blood flow, depletes the blood supply reserve, disrupts the BBB, and reduces the regenerative potential of the brain. These effects secondarily increase ischemia and the attending neurodegeneration, thus closing the pathological vicious circle [56]. Therefore, neurodegeneration in patients with VP will be secondary in nature because of the effect of hypoxia on all the nervous tissue: not only on neurons, but on glial cells as well.

The effect of chronic ischemia on nervous tissue is primarily characterized by the occurrence of oxidative stress and inflammation. Disturbance in the redox state of cells resulting from ischemia can cause toxic effects through the formation of peroxides and free radicals that damage almost all cell components, including proteins, lipids, and DNA [8]. Chronic ischemia also leads to mitochondrial dysfunction and inhibition of protein synthesis, which can disrupt the balance between antioxidants and reactive oxygen species. Such oxidative damage to vascular endothelial cells, glia, and neurons may further impair the vascular function and intercellular interactions between neurons, astrocytes, and microvessels, followed by a drop in cerebral perfusion [57].

Permanent bilateral carotid artery stenosis is an experimental model of chronic ischemia in laboratory animals. In particular, the rat model was used to show the emergence of synaptic dysfunction in the hippocampus associated with cognitive impairment [58]. Reduced pyruvate dehydrogenase level and increased oxidative stress were also observed, indicating that mitochondrial energy deficiency affects memory [59].

Extensive damage to the white matter of the brain is also observed in chronic arterial occlusion. The degree of ischemic damage positively correlates with white matter involvement in the pathological process, the greatest structural damage being observed within the corpus callosum [60]. White matter dysfunction is associated with activation of glial cells: on the one hand, glial cells are activated immediately in response to damage to white matter by oxidative stress, while on the other hand, damage to the BBB facilitates penetration of immune cells and release of an enormous number of proinflammatory cytokines, as well as serine proteases, matrix metalloproteinase 2, elastase, and collagenase [55]. These released components damage the extracellular matrix and cause a remodeling of vascular walls, which eventually leads to damage and further destruction of the BBB and white matter. Axonal injury (of the white matter) caused by destruction of afferent neuronal connections, or their retrograde injury, will be followed by apoptosis of neurons *per se*. Furthermore, pro-inflammatory cytokines infiltrating the white matter disrupt growth factor signaling, inducing the state of “neurotrophin resistance”. Loss of trophic support can impede proliferation, migration, and differentiation of oligodendrocyte progenitor cells as well as impair white matter repair. Glial scars will develop at the damage site [61]. Hence, chronic ischemia is characterized by atrophy of cortical neurons and a reduced volume of the entire cerebral cortex, as well as edema and damage to the white matter in the form of demyelination, apoptosis of oligodendrocytes and atrophy, as well as glial proliferation in the form of astrocytogliosis [62].

In other studies, endothelial dysfunction worsening the ability of vessels to respond to changes in cerebral hemodynamics is reported to play the leading role in the pathophysiology of chronic ischemia. The resulting impairment of neurovascular coupling leads to transient or chronic cerebral hypoperfusion [63]. Endothelial cells are capable of recognizing immune signals and expressing adhesion molecules (P- and E-selectin, intercellular adhesion molecules, vascular cell adhesion, etc.), which recognize certain molecules on circulating immune cells, leading to transmigration of these cells into the brain [64]. Cytokines produced by perivascular macrophages, endothelium, and glia regulate the expression of adhesion molecules, other cytokines and chemokines, as well as promote leukocyte trafficking across the BBB [65]. This process is important for both immune surveillance in the normal brain and the brain’s immune response to injury. Moreover, oxidative stress-induced endothelial dysfunction may cause the release of the vascular endothelial growth factor (VEGF) and prostanoids, which promote endothelial leakage of active substances and inflammation [66]. Extravasation of plasma proteins also causes vascular inflammation, oxidative stress, perivascular edema, and axonal demyelination. However, it is most likely that the described processes (arterial occlusion, impaired cerebral microcirculation, BBB damage, and endothelial dysfunction) run simultaneously and mutually exacerbate each other’s effects.

**Potential markers of vascular parkinsonism in metabolomic studies of various cerebrovascular diseases**

There are very few metabolomic studies of vascular parkinsonism; so, this review discusses data on the metabolomic profile in cerebrovascular diseases as a basis for the development of VP. Numerous metabolites are regarded as biomarkers of cerebrovascular parkinsonism in experiments: ranging from low-molecular-weight (amino acids, nucleotides and other products of impaired metabolic pathways) to groups of proteins responsible for a number of body functions (*Table 2*).

**Table 2:** Metabolomic markers of various cerebrovascular diseases related to the development of vascular parkinsonism

Biological matrix	Cerebrovascular disease/condition	Found biomarkers	Reference
Blood plasma	White matter hyperintensity in MRI scans	Sphingomyelin 38:1 (SM 38:1), ceramide 34:1 (Cer 34:1)	[67]
Blood serum	Small vessel disease	Creatine, fatty acid 18:2(OH), sphingomyelin (d18:2/24:1), N1-acetylspermidine, N-acetylputrescine, isoleucine, creatinine, creatine, cytosine, 5’-methylthioadenosine	[68]
Blood plasma	Large artery atherosclerosis (LA), small vessel disease (SVD)	Cer (d36:3), Cer (d34:2), Cer (d38:6) (for LA); SM (d34:1), Cer (d34:2), Cer (d36:4) (for SVD)	[69]
Blood serum	Vascular dementia	7α-hydroxycholesterol, primary bile acids	[70]
Blood serum	Vascular dementia, mixed dementia	L-arginine, L-arginine/asymmetric dimethylarginine ratio, L-arginine/nitric acid pathway	[71]
Blood serum	Vascular dementia	Dihydroxybutanoic, docosapentaenoic and uric acid	[72]
Blood plasma	Chronic cerebral ischemia	Proteins SERPINF2, HRG, KNG1, APCS, C1R, C5, AGT, PROS1, ITIH1, etc.	[73]
Cerebral cortex	Vascular dementia	Proteins SOD1, NCAM, and ATP5A	[74]

Many metabolomic studies aim to establish a connection between the known structural changes in patients with CVDs and the metabolic pathways that lead to them. For example, white matter hyperintensity is a frequent sign of CVD in MRI images. One study of plasma metabolites from patients whose MRI scans had shown white matter hyperintensity but who had not been diagnosed with ischemic stroke or transient ischemic attacks revealed an association between sphingolipids and the severity of the changes in MRI scans [67]. Two such metabolites, sphingomyelin 38:1 (SM 38:1) and ceramide 34:1 (Cer 34:1), were identified using HPLC/MS. Both ceramides and sphingomyelins are components of lipid envelopes, which play a crucial role in maintaining the myelin structure. It was hypothesized that these molecules can be specific biomarkers of white matter damage and can also be indicative of CVD progression based on the degree of white matter involvement in the pathological process

Data from another study employing a combination of HPLC/MS and NMR showed higher levels of creatine, unsaturated acid 18:2(OH), and sphingomyelin (d18:2/24:1), which were associated with a larger number of lacunae, white matter damage in MRI scans, and worsened cognitive performance [68]. Elevated levels of seven amino acids and nucleotides (N1-acetylspermidine, N-acetylputrescine, isoleucine, creatinine, creatine, cytosine, and 5’-methylthioadenosine) were found to be associated with the emergence of similar lesions. Low serum levels of several sphingomyelins and glycerophospholipids turned out to be markers of more severe white matter damage, brain atrophy, and cognitive impairment.

You et al. detected 276 sphingolipids, including 39 ceramides (Cer), three ceramide phosphates, 72 glycosphingolipids, and 162 sphingomyelins (SM), in patients’ plasma; their levels differed from those in the control group [69]: the levels of Cer (d36:3), Cer (d34:2), Cer (d38:6), etc. were elevated in patients with large artery atherosclerosis; the levels of SM (d34:1), Cer (d34:2), Cer (d36:4), etc. were increased in patients with age-related small vessel disease. The levels of Cer (d36:4) and SM (d34:1) in patients with age-related small vessel disease were elevated compared to those in patients with large artery atherosclerosis.

Changes in the lipid profile may play a role in the pathogenesis of CVDs. Hence, a reduced serum cholesterol level was reported to be associated with the emergence of neuroimaging markers of vascular dementia, while statin-induced pharmacological reduction in cholesterol levels can be associated with an increased risk of vascular dementia in males [70]. The low serum levels of 7α-hydroxycholesterol and primary bile acids are associated with a higher degree of cerebral amyloid deposition, significant white matter damage, and more rapid brain atrophy. The study used a combination of such methods as targeted plasma metabolomic profiling, positron emission tomography, brain MRI, and pharmacoepidemiologic analysis.

The *L*-arginine/nitric oxide pathway was shown to be altered in people with dementia [71]. Thus, plasma specimens from patients with vascular dementia, Alzheimer’s disease, and mixed dementia were studied using targeted metabolomic screening by HPLC-MS. All the types of dementia were found to be associated with reduced levels of L-arginine, asymmetric dimethylarginine and L-citrulline, as well as with reduced L-arginine/asymmetric dimethylarginine ratio. Meanwhile, the level of *L*-arginine and the* L*-arginine/asymmetric dimethylarginine ratio differentiated vascular dementia and Alzheimer’s disease. Changes in the levels of these metabolites were indicative of structural brain alterations and correlated with the severity of the cognitive impairment.

Metabolomic profiling can also predict the development of vascular dementia. Thus, the role of dihydroxybutanoic, docosapentaenoic, and uric acids in a 5-year progression of the disease was demonstrated [72]. Plasma specimens from patients with vascular dementia and Alzheimer’s disease, as well as subjects without diagnosed dementia but with a potential to develop age-related dementia, was analyzed by HPLC-MS in a prospective study. The levels of the aforementioned substances were elevated in the group of patients with both types of dementia and in incident cases within five years before the onset of dementia.

Along with analyzing the low-molecular-weight metabolites, metabolomic studies of the plasma protein profile of patients with CVDs were also conducted. Thus, a group comprising 44 proteins involved in the blood coagulation pathway (SERPINF2, HRG, KNG1, etc., a total of ten proteins), in activation of innate immunity (APCS, C1R, C5, etc., a total of 13 proteins), and in regulation of the activities of hydrolases AGT, PROS1, ITIH1, etc. was revealed [73]. The observed shift in the weights of functional protein clusters is supposedly attributable to the activation of compensatory mechanisms aiming to maintain a homeostasis.

Another study showed upregulated expression of the SOD1 and NCAM proteins and downregulated expression of the ATP5A protein in patients with vascular dementia, an indication of nervous tissue hypometabolism and vascular insufficiency, along with inflammation [74]. The increased SOD1 level, as well as a trend toward increasing levels of iron uptake proteins (FTL and FTH1) can be indicative of an oxidative imbalance that is accompanied by iron metabolism disorders.

**Metabolomic studies of cerebrovascular diseases in experimental models**

Metabolomic studies of CVDs are also conducted on experimental models. Desorption electrospray ionization mass spectrometry was employed to illustrate lipid distribution in the rat brain in a similar model. In the experimental specimens, reduced levels of dihomo- γ-linolenic, stearic, arachidonic, docosahexaenoic, and hydroxyeicosatetraenoic acids, as well as ethanolamine glycerophosphate, were observed in the entire brain, and especially in the hippocampus. The reduced levels of these substances in patients with CVDs can be attributed to the anti-inflammatory properties of some of them (e.g., dihomo-γ-linolenic acid), as well as to the loss of nervous tissue *per se*. In the corpus callosum, the signal intensities of three glycerophospholipids (LMGP06010075, LMGP00000053, and LMGP0601010168) and sulfatide, which are myelin components, were reduced [75].

In this section, we would also like to summarize some results of a proteomic analysis, or highthroughput protein analysis. In proteomics, Tukacs et al. revealed changes in the protein profile in rats with stepwise bilateral occlusion of the common carotid artery. They identified a large number of proteins whose regulation in the occipital lobe of the cortex differs from that in the frontal cortex and hippocampus [76]. The altered proteins possess the functions associated with cytoskeletal organization and energy metabolism. Thus, the expression of proteins involved in the citric acid cycle and electron transport chain (fructose-bisphosphate aldolase C, ATP synthase alpha, isocitrate dehydrogenase, NADH dehydrogenase, etc.) was found to be downregulated.

## CONCLUSIONS

It is obvious that the commonality of the neurodegeneration concept not only makes clinical sense, but it also implies common metabolic pathways and metabolomic targets. Searching for markers of individual neurodegenerative processes, such as PD and ischemic neurodegeneration, is of particularly specific interest, as it encompasses such clinical concepts as early manifestations, prediction of the disease course, diagnostic criteria, and personalization of the therapeutic approach. For PD, such markers are metabolites of amino acids, acylcarnitines, fatty acids, bile acids; for CVDs, these markers are the proteins involved in the coagulation pathway or the regulation of the immune response.

The capabilities of a metabolomic analysis open up prospects for the clinical diagnostics of neurodegenerative diseases, shortening the time between admission and diagnosis, which currently takes ten years.

A review of the literature focusing on the search for metabolomic markers of Parkinson’s disease (PD) and vascular parkinsonism (VP) yields conflicting results. All the available evidence indicates that studying metabolomic abnormalities in a single humoral environment alone is of little practical value. In order to identify both general signs of primary neurodegeneration (characteristic of PD) and the ischemic nature (as in the case of VP), as well as their differential markers, one needs to simultaneously examine blood plasma and cerebrospinal fluid. Differences in the neurotransmitter activity of the subcortical structures associated with PD and VP infer that there are significant differences in metabolites characteristic of each of these conditions. In patients with PD, the neurons of the substantia nigra, which produce dopamine, are primarily affected. Meanwhile, VP is characterized by damage to the globus pallidus and putamen, which function at the expense of other mediators such as GABA, glutamate, choline, etc. Further research should avail itself of the benefits of widespread use of available diagnostic tests such as whole blood or dried serum spot analysis.

Furthermore, the study of the range of low-molecular- weight markers is supposed to help one decipher the cascade of metabolic disorders, the stage of their involvement, and their effect on the clinical picture of motor as well as cognitive mental disorders, with allowance for patient sex. This approach will allow one to assess the significance of metabolic failures in the pathogenesis of two different types of neurodegenerative disorders.

## Abbreviations

PD: Parkinson’s disease
HPLC/MS: high-performance liquid chromatography-mass spectrometry
BBB: blood-brain barrier
LC/MS: liquid chromatography-mass spectrometry
MRI: magnetic resonance imaging
CSF: cerebrospinal fluid
VP: vascular parkinsonism
CNS: central nervous system
CVD: cerebrovascular disease
